# Factors associated with treatment resumption and long-term outcomes after ICU discharge in patients with hematological malignancies

**DOI:** 10.1016/j.aicoj.2026.100152

**Published:** 2026-09-08

**Authors:** Ondine Messéant, Mathilde Guégan, Benoît Painvin, Adel Maamar, Jean-Baptiste Mear, Jean Reignier, Emmanuel Canet, Jean-Marc Tadié, Soraya Benguerfi

**Affiliations:** a Lymphoid Hematological Diseases Department, Henri Mondor University Hospital, AP-HP, Créteil, France; b Hematology Department, Rennes University Hospital, Rennes, France; c Medical Intensive Care Unit, Rennes University Hospital, Rennes, France; d Medical Intensive Care Unit and Movement-Interactions-Performance (MIP) Research Unit 4334, Nantes University Hospital, Nantes, France; e Medical Intensive Care Unit, Nantes University Hospital, Nantes, France; f SITI Laboratory, UMR U1236, INSERM, Rennes University and French Blood Agency (EFS), Rennes University Hospital, Rennes, France; g Rennes Clinical Investigations Center (CIC1414), INSERM, Rennes University Hospital, Rennes, France

**Keywords:** Malignancies, Intensive care unit, Cancer treatment, Outcome, Hematological malignancies

## Abstract

**Purpose:**

Post-intensive care syndrome may result in persistent functional impairments that may jeopardize the feasibility of anticancer therapy in patients with hematological malignancies (HMs), thereby adversely affecting long-term survival. Our purpose was to identify factors associated with anticancer therapy resumption after ICU discharge and to assess five-year survival.

**Methods:**

This single-center retrospective observational study included consecutive adults with HMs admitted to intensive care unit (ICU) between 2014 and 2019. Multivariable analyses were performed to identify factors associated with anticancer treatment resumption and with survival.

**Results:**

We included 252 patients. The most common malignancies were aggressive lymphoma (n = 71, 28.2%), acute leukemia (n = 47, 18.7%), and multiple myeloma (n = 42, 16.7%). Performance status worsened significantly during the ICU stay (median change, −1.0; 95%CI, −1.0 to −1.0; *P* < 0.001). Of the 171 patients (67.9%) discharged alive from the ICU, 119 had an indication for further anticancer therapy; among them, 106 (89.1%) resumed treatment, although modifications of the regimen were required in 20/103 (19.4%). By multivariable analysis, cardiovascular disease was independently associated with a lower likelihood of treatment resumption (hazard ratio [HR], 0.41; 95%CI, 0.20–0.84; *P* = 0.014) and diagnosis of the HM during the ICU stay with a higher likelihood of treatment resumption (HR, 3.03; 95%CI, 1.53–6.01; *P* = 0.002). Survival rates after ICU admission were 50.0% at six months, 46.0% at one year, 41.3% at two years, 38.9% at three years, and 36.1% at five years. Factors independently associated with a lower risk of five-year mortality were infection (HR, 0.54; 95%CI, 0.33–0.87; *P* = 0.011), HM diagnosed during the ICU stay (HR, 0.31; 95%CI, 0.12–0.81; *P* = 0.018), and classification as other HMs (HR, 0.40; 95%CI, 0.19–0.86; *P* = 0.019).

**Conclusions:**

In patients with HMs, ICU survival was substantial and most deaths occurred during the ICU stay or within the first post-ICU year. Most survivors were able to resume anticancer therapy despite exhibiting functional decline. Long-term outcomes in survivors were primarily related to the nature of the underlying disease and to cardiovascular comorbidities. Both the oncological prognosis and the cardiovascular status should be considered when making intensive-care decisions.

## Introduction

Therapeutic advances over the past decades have transformed the management of hematological malignancies (HMs), even in elderly and frail patients. Novel agents, intensified regimens, and cellular therapies have improved survival but carry substantial toxicity [1]. Patients remain exposed to life-threatening complications related to the disease and its treatment, both of which induce immunosuppression [2,3]. About 14% of patients with HMs require intensive-care-unit (ICU) admission within the first year after the diagnosis [4]. Short-term outcomes have improved in critically ill patients with malignancies [5]. Consequently, ICU admission policies have been broadened, and a growing proportion of ICU beds is occupied by patients with malignancies [6].

Few studies have focused on the long-term outcomes of patients with HMs after ICU discharge. Moreover, the populations and endpoints vary across studies, and data beyond one year are scarce [7,8]. Post-intensive care syndrome — defined as new or worsened physical, cognitive, and psychological impairments that may persist for years — may compromise the ability to resume or continue anticancer therapy [9]. Persistent organ dysfunction and functional decline may also require a decrease in treatment intensity, thereby adversely affecting long-term survival. Thus, in a 2024 single-center retrospective observational study, 42% of ICU survivors with acute myeloid leukemia or aggressive B-cell lymphoma required changes in their anticancer treatment, which were associated with worse one-year progression-free and overall survival [7].

This single-center retrospective observational cohort study in patients with HMs who required ICU admission investigated outcomes over the next five years. The primary objective was to identify factors associated with anticancer treatment resumption after ICU discharge. The secondary objective was to assess long-term survival, up to five years after ICU admission.

## Methods

### Study design and population

We conducted a single-center retrospective observational cohort study in a 30-bed general medical ICU of a French, university-affiliated hospital. All adults with HMs admitted to the ICU between 2014 and 2019 were eligible. In patients with multiple ICU admissions during the study period, we considered only the first ICU stay. The study was approved by our institutional ethics committee (#20.02).

### Data collection

The clinical data were extracted from the electronic medical records. Data at ICU admission were demographics and the Eastern Cooperative Oncology Group performance status (ECOG PS) [10], medical history, severity of critical illness as assessed by the Simplified Acute Physiology Score II (SAPS II) [11], and presence of shock and/or infection. Cardiovascular risk factors were defined as diabetes mellitus, dyslipidemia, hypertension, overweight or obesity, and obstructive sleep apnea [12]. Cardiovascular disease was defined as any established cardiovascular condition such as ischemic heart disease, peripheral arterial disease, abdominal aortic aneurysm, atrial fibrillation, or other documented cardiovascular disorder. Chronic alcohol abuse was alcohol consumption exceeding the low-risk amount recommended in France (i.e., >10 standard drinks per week, >2 standard drinks per day, or no alcohol-free days during the week) [13]. Septic shock was defined according to Sepsis-3 criteria [14].

We recorded the following characteristics of the HM: date of diagnosis, whether the diagnosis was made during the ICU stay, HM type, HM status at ICU admission (remission, new diagnosis, relapse diagnosis, progressive despite ongoing treatment, non-progressive with ongoing treatment, or watchful waiting), previous anticancer treatments (chemotherapy, radiotherapy, immunotherapy, and/or targeted therapy), autologous or allogeneic hematopoietic stem-cell transplantation (HSCT), number of prior treatment lines, and whether anticancer therapy was ongoing at ICU admission. The HMs were categorized into five predefined groups: allogeneic HSCT, acute leukemia, aggressive lymphoma, multiple myeloma, and other HMs. Anticancer treatment was considered ongoing if administered within two months before ICU admission.

ICU-related data collected for the study were length of stay, reason for admission, highest number of organ failures [15,16] as assessed by the Sequential Organ Failure Assessment (SOFA) score [17] and excluding thrombocytopenia (which may be treatment-related or cancer-related), highest number of organ-support interventions, use of invasive or noninvasive mechanical ventilation, vasopressor support, renal replacement therapy, extracorporeal membrane oxygenation, presence of acute respiratory distress syndrome, and administration of anticancer treatment during the ICU stay. Vital status, cause of death and HM status at death were recorded. In ICU survivors, the ECOG performance status (PS) at ICU discharge was recorded.

For patients in whom further anticancer treatment was indicated after ICU discharge, collected data on post-ICU systemic or local anticancer treatments were type of treatment (chemotherapy, radiotherapy, immunotherapy, and/or targeted therapy), modifications of the initial treatment plan (drug changes and/or dosage reductions), tumor response (stable disease, partial response, or complete response at the time of the oncology assessment after anticancer treatment resumption), and date of first disease progression after anticancer treatment resumption. To determine whether further anticancer treatment was indicated after ICU discharge, two hematologists (OM and MG) independently reviewed the medical records of all ICU survivors, each blinded to the other's assessment. In accordance with institutional practice, all decisions regarding anticancer treatment initiation or resumption were discussed and validated during multidisciplinary hematology tumor board meetings. Whenever a documented tumor board decision was available, it was considered the reference standard for determining treatment indication. In the absence of a documented tumor board decision, treatment indication was retrospectively adjudicated by the two hematologists based on the available clinical and hematological information. In case of disagreement, review by a third senior hematologist (JBM) was planned; however, no disagreements occurred. Intensivists were not routinely involved in multidisciplinary hematology tumor board meetings but could be invited to participate in complex clinical situations, particularly in patients with major comorbidities, after recent critical illness, or when anticancer therapies with a potentially high risk of toxicity were being considered.

Poor PS was defined as an ECOG PS of 3 (capable of only limited self-care; confined to bed or chair more than 50% of waking hours) or 4 (completely disabled; totally confined to bed or chair) [10]. Changes in PS were assessed as the difference between performance before and after the ICU stay. The pre-ICU PS was the ECOG PS determined during the most recent hematology visit, within three months before ICU admission. The post-ICU ECOG PS was assessed within one week following ICU discharge.

### Statistical analysis

Descriptive statistics were used to describe the study population. We compared patients who did vs. did not receive anticancer treatment after ICU discharge. For categorical variables, we applied the chi-square test or Fisher’s exact test, as appropriate. Continuous variables were compared using Student’s *t*-test or the Wilcoxon-Mann Whitney test, as appropriate. In ICU survivors, the difference between pre-ICU and post-ICU ECOG PS was assessed using the paired Wilcoxon signed-rank test.

The analysis of treatment resumption was restricted to ICU survivors with an indication for further anticancer treatment after ICU discharge. Variables associated with anticancer treatment resumption after ICU discharge by univariable analysis (*P* < 0.10) were entered into a multivariable Fine and Gray competing risks model, after assessment of collinearity. Time zero was defined as the date of ICU discharge. The event of interest was resumption of anticancer treatment, while death before treatment resumption was considered a competing event, as it permanently precluded the occurrence of the event of interest. Patients who were alive without treatment resumption at the last available follow-up were censored at the date of last contact.

Survival analyses were performed. Overall survival was defined as the time from ICU admission to death from any cause. Variables associated with survival by univariable analysis (*P* < 0.10) were included in a multivariable Cox proportional hazards model, after verification of collinearity and proportional hazards assumptions. Hazard ratios (HRs) with their 95% confidence intervals (95%CI) were computed. To further evaluate the relationship between ICU severity and outcomes, the highest number of organ failures and organ supports were modeled both as continuous variables and as predefined categorical variables (0–1, 2, and ≥3) in the analyses of anticancer treatment resumption and survival. HM type was included as a categorical variable, with allogeneic HSCT as the reference category. Anticancer treatment resumption was intentionally excluded from the multivariable survival model to minimize potential confounding. Survival probabilities according to HM type were estimated using the Kaplan-Meier method.

All statistical tests were two-sided and *P* values ≤0.05 were taken to indicate significant differences. All analyses were performed using the R program (https://www.r-project.org)

## Results

### Overall population characteristics

Between 2014 and 2019, 252 patients with HMs were admitted to our ICU. Table 1 reports their main baseline and ICU-related characteristics. Before ICU admission, 129 patients (51.2%) had a poor PS (ECOG PS 3–4).

**Table 1:** Baseline and ICU-related characteristics of the overall population (n = 252).

Characteristics	Overall population (n = 252)
Male sex, n (%)	149 (59.1)
Age at ICU admission, years, median [IQR]	61 [49−69]
ECOG PS 3−4 before ICU admission^a^, n (%)	129 (51.2)
SAPS II, median [IQR]	48 [35−64]
Shock, n (%)	119 (47.2)
Types of shock, n (%)	
Septic	91 (76.5)
Hypovolemic	8 (6.7)
Anaphylactic	1 (0.8)
Hemorrhagic	7 (5.9)
Cardiogenic	12 (10.1)
Infection, n (%)	168 (66.7)
Infection sites, n (%)	
Respiratory	81 (48.2)
Cutaneous	3 (1.8)
Urinary	10 (6.0)
Gastrointestinal	43 (25.6)
Catheter-related bloodstream infection	8 (4.8)
Fungemia	1 (0.6)
Other	22 (13.1)
Medical history^b^, n (%)	
History of solid malignancy	30 (11.9)
History of another HM	103 (40.9)
Chronic pulmonary disease	36 (14.3)
Cardiovascular disease	52 (20.6)
Cardiovascular risk factors	103 (40.9)
Cirrhosis	4 (1.6)
Stroke and/or transient ischemic attack	7 (2.8)
Chronic renal failure	16 (6.3)
Chronic alcohol abuse	10 (4.0)
Time from HM diagnosis to ICU admission, days, median [IQR]	362.5 [89.3–1642.0]
HM diagnosed in the ICU, n (%)	30 (11.9)
HM classification for analysis, n (%)	
Aggressive lymphoma	71 (28.2)
Acute leukemia	47 (18.7)
Multiple myeloma^c^	42 (16.7)
Allogeneic hematopoietic stem cell transplant	35 (13.9)
Others	57 (22.6)
HM types, n (%)	
Myeloid malignancies	
Acute myeloid leukemia	48 (19.0)
Myelodysplastic syndrome	9 (3.6)
Myeloproliferative syndrome	8 (3.2)
Myelodysplastic/myeloproliferative neoplasm	6 (2.4)
Multiple myeloma^c^	43 (17.1)
Aggressive B-cell lymphoid malignancies	
Diffuse large B-cell lymphoma	31 (12.3)
Acute lymphoblastic leukemia / Lymphoblastic lymphoma	19 (7.5)
Burkitt lymphoma	11 (4.4)
Mantle-cell lymphoma	6 (2.4)
Indolent B-cell malignancies	
Follicular lymphoma	10 (4.0)
Chronic lymphocytic leukemia / Small lymphocytic lymphoma	20 (7.9)
Waldenström macroglobulinemia	5 (2.0)
Hairy-cell leukemia	3 (1.2)
Other low-grade B-cell lymphomas	3 (1.2)
Other lymphoid malignancies	
T-cell lymphoma	15 (6.0)
Hodgkin lymphoma	15 (6.0)
Anticancer treatments received before ICU admission, n (%)	
Radiotherapy	28 (11.1)
Chemotherapy	198 (78.6)
Immune checkpoint inhibitors	83 (32.9)
Targeted therapy	17 (6.7)
HSCT before ICU admission, n (%)	
Allogeneic HSCT	34 (13.7)
Autologous HSCT	37 (14.9)
Autologous followed by allogeneic HSCT	1 (0.4)
Time from allogeneic HSCT to ICU admission, days, median [IQR]	51.0 [10.5–290.0]
Number of anticancer treatment lines before ICU admission, median [IQR]	1 [1–2]
Length of stay in the ICU, days, median [IQR]	3.0 [2.0–7.0]
Reason for ICU admission, n (%)	
Septic shock	65 (25.8)
Infection without shock	25 (9.9)
Acute respiratory failure	72 (28.6)
Metabolic disorders	4 (1.6)
Altered mental status	16 (6.3)
Acute kidney injury	4 (1.6)
Hemorrhagic shock	4 (1.6)
Liver transplantation	2 (0.8)
Tumor lysis syndrome	9 (3.6)
Cardiac arrest	6 (2.4)
Hemoptysis	3 (1.2)
Status epilepticus	4 (1.6)
Undifferentiated shock	10 (4.0)
Abnormal complete blood count	7 (2.8)
Compressive mediastinal mass	2 (0.8)
Cardiogenic shock	3 (1.2)
Postoperative monitoring	3 (1.2)
Other	13 (5.2%)
Highest number of organ failures, median [IQR]	2 [1–3]
Highest number of organs supported, median [IQR]	1 [1–2]
Invasive mechanical ventilation, n (%)	108 (43.0)
Noninvasive ventilation, n (%)	19 (7.6)
High-flow nasal cannula oxygen therapy, n (%)	19 (7.5)
Vasopressor support, n (%)	133 (53.0)
Renal replacement therapy, n (%)	37 (14.7)
V-A ECMO, n (%)	1 (0.4)
V-V ECMO, n (%)	2 (0.8)
Acute respiratory distress syndrome, n (%)	38 (15.1)
Anticancer treatment during ICU stay, n (%)	46 (18.3)
Type of anticancer treatment in the ICU, n (%)	
Chemotherapy	28 (60.9)
Targeted therapy	1 (2.2)
Immunotherapy	2 (4.3)
Chemotherapy and immunotherapy	7 (15.2)
Chemotherapy and targeted therapy	1 (2.2)
Corticosteroids	7 (15.2)
ECOG PS 3–4 after ICU discharge^a^, ^d^, n (%)	118 (71.5)

ECOG PS: Eastern Cooperative Oncology Group Performance Status; SAPS II: Simplified Acute Physiology Score version II; HM: hematological malignancy; ICU: intensive care unit; HSCT: hematopoietic stem-cell transplantation; V-A ECMO: veno-arterial extracorporeal membrane oxygenation; V-V ECMO: veno-venous extracorporeal membrane oxygenation.

a An ECOG PS of 3 or 4 defined poor performance status.

b Some patients had a history of more than one of the listed conditions.

c One patient with multiple myeloma was classified in the allogeneic HSCT group for the statistical analyses, resulting in 42 cases in the analytical classification versus 43 in the descriptive HM types.

d Available for 165 of the 171 ICU survivors.

The most common HMs were aggressive lymphoma (n = 71, 28.2%), acute leukemia (n = 47, 18.7%), and multiple myeloma (n = 42, 16.7%). A history of allogeneic HSCT was present in 35 (13.9%) patients. At ICU admission, 51/247 (20.6%) patients were in remission, 63/247 (25.5%) had newly diagnosed disease, and 18/247 (7.3%) were experiencing a relapse. In addition, 85/247 (34.4%) patients were receiving active treatment and had non-progressive disease, 17/247 (6.9%) patients had progressive disease despite receiving active treatment, and 13/247 (5.3%) patients were being managed by watchful waiting. Anticancer treatment was ongoing at ICU admission in 160 (63.5%) patients. The main reasons for ICU admission were acute respiratory failure (n = 72, 28.6%) and septic shock (n = 65, 25.8%).

During the ICU stay, life-sustaining treatments were withdrawn or withheld in 35/250 (14.0%) patients.

Of the 171 patients discharged alive from the ICU, 118/165 (71.5%) patients had a poor PS (ECOG PS 3–4). PS was significantly worse after than before the ICU stay, the median decrease being one ECOG point (95%CI, -1.0 to -1.0; *P* < 0.001).

### Anticancer treatment resumption after ICU discharge

**Fig. 1:**
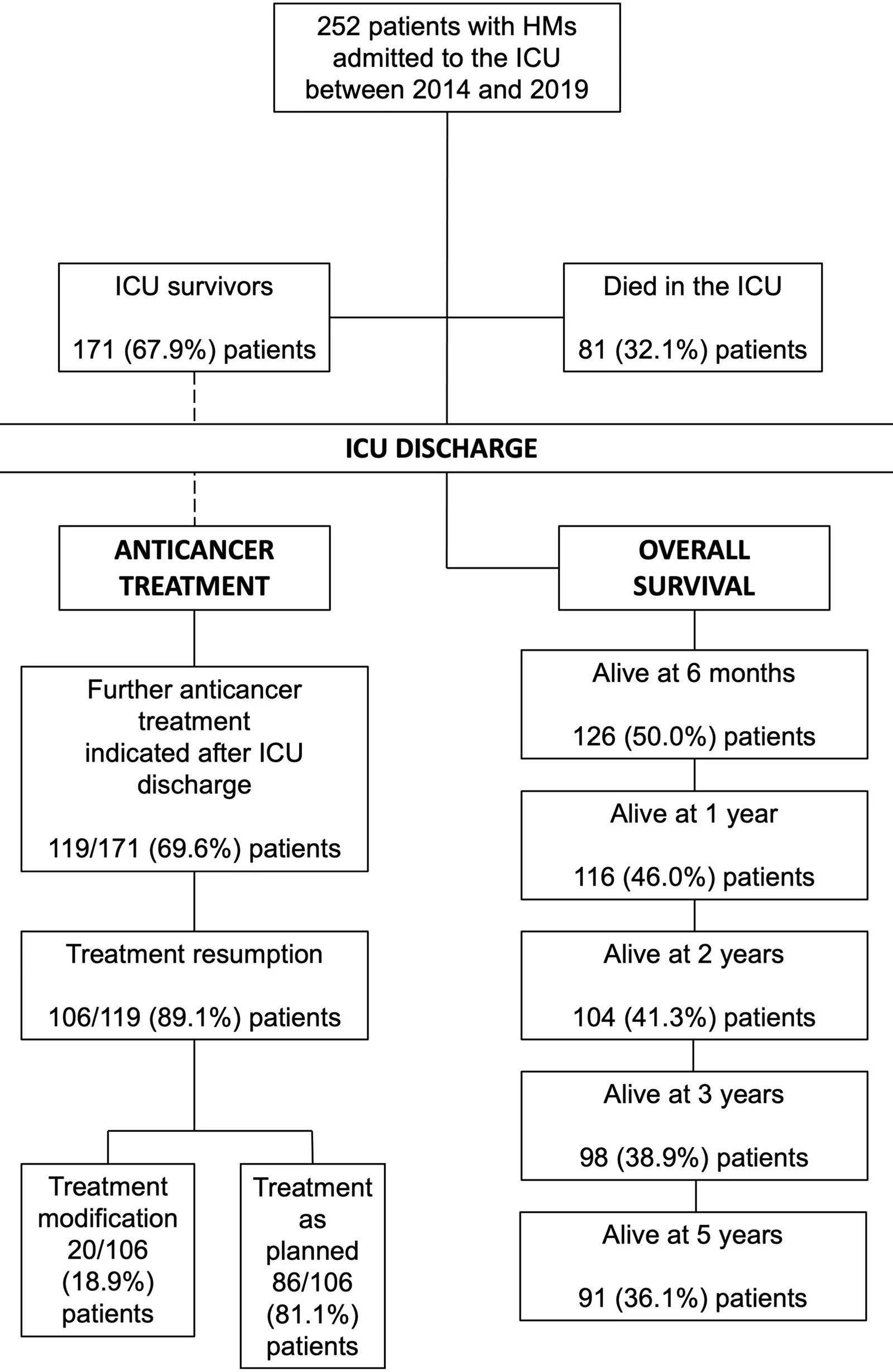
Trajectory of patients from ICU admission through ICU survival, post-ICU treatment decisions, and five-year outcomes.

Of the 171 ICU survivors, 119 (69.6%) patients had indications for further anticancer treatment. Table 2 reports their baseline and ICU-related characteristics. Of the 119 patients, 106 (89.1%) did and 13 (10.9%) did not receive anticancer treatment after ICU discharge. Fig. 1 shows the proportions of patients given anticancer treatment.

**Table 2:** Baseline and ICU-related characteristics of patients with indications for further anticancer treatment after ICU discharge.

Characteristics	Patients requiring anticancer treatment after ICU discharge (n = 119)	No anticancer treatment after ICU discharge (n = 13)	Anticancer treatment after ICU discharge (n = 106)
Male sex, n (%)	70 (58.8)	11 (84.6)	59 (55.7)
Age at ICU admission, years, median [IQR]	60 [49−67]	65 [60−65]	59 [47−67]
ECOG PS 3 or 4 before ICU admission^a^, n (%)	52 (43.7)	8 (61.5)	44 (41.5)
Detailed ECOG PS distribution			
0	5 (4.2)	0 (0.0)	5 (4.8)
1	61 (51.7)	5 (38.5)	56 (53.3)
2	40 (33.9)	6 (46.2)	34 (32.4)
3	12 (10.2)	2 (15.4)	10 (9.5)
4	0 (0.0)	0 (0.0)	0 (0.0)
SAPS II, median [IQR]	40 [32−53]	47 [36−55]	39 [30−53]
Shock, n (%)	38 (31.9)	5 (38.5)	33 (31.1)
Infection, n (%)	69 (58.0)	8 (61.5)	61 (57.5)
Medical history^b^, n (%)			
History of solid malignancy	12 (10.1)	2 (15.4)	10 (9.4)
History of another HM	44 (37.0)	7 (53.8)	37 (34.9)
Chronic pulmonary disease	10 (8.4)	3 (23.1)	7 (6.6)
Cardiovascular disease	21 (17.6)	8 (61.5)	13 (12.3)
Cardiovascular risk factors	41 (34.5)	8 (61.5)	33 (31.1)
Cirrhosis	2 (1.7)	1 (7.7)	1 (0.9)
Stroke and/or transient ischemic attack	3 (2.5)	1 (7.7)	2 (1.9)
Chronic renal failure	7 (5.9)	0 (0.0)	7 (6.6)
Chronic alcohol abuse	4 (3.4)	0 (0.0)	4 (3.8)
Time from HM diagnosis to ICU admission, days, median [IQR]	184 [31−1265]	730 [109–996]	131 [26−1278]
HM diagnosed in the ICU, n (%)	21 (17.6)	0 (0.0)	21 (19.8)
HM classification for analysis, n (%)			
Allogeneic HSCT	4 (3.4)	1 (7.7)	3 (2.8)
Acute leukemia	32 (26.9)	0 (0.0)	32 (30.2)
Aggressive lymphoma	41 (34.5)	7 (53.8)	34 (32.1)
Multiple myeloma	21 (17.6)	1 (7.7)	20 (18.9)
Other	21 (17.6)	4 (30.8)	17 (16.0)
HM status at ICU admission, n (%), missing data = 1			
Remission	7 (5.9)	2 (15.4)	5 (4.8)
New diagnosis	44 (37.3)	1 (7.7)	43 (41.0)
Progression without treatment / relapse	8 (6.8)	3 (23.1)	5 (4.8)
Watchful waiting	0 (0.0)	0 (0.0)	0 (0.0)
On anticancer treatment and non-progressive	52 (44.1)	7 (53.8)	45 (42.9)
On anticancer treatment and progressive	7 (5.9)	0 (0.0)	7 (6.7)
Number of anticancer treatment lines before ICU admission, median [IQR]	1 [1,2]	2 [1,2]	1 [1,2]
Ongoing anticancer treatment, n (%)	86 (72.3)	11 (84.6)	75 (70.8)
Length of stay in the ICU, days, median [IQR]	3 [2–6]	6 [3–8]	3 [2–5]
Highest number of organ failures, median [IQR]	1 [1,2]	2 [1,2]	1 [1,2]
Highest number of supported organs, median [IQR]	1 [1,1]	1 [1,2]	1 [0−1]
Acute kidney injury, n (%)	46 (38.7)	8 (61.5)	38 (35.8)
KDIGO-defined acute kidney injury stage			
1	22 (47.8)	4 (50.0)	18 (47.3)
2	11 (23.9)	3 (37.5)	8 (21.1)
3	13 (28.3)	1 (12.5)	12 (31.6)
Invasive mechanical ventilation, n (%)	22 (18.5)	3 (23.1)	19 (17.9)
Noninvasive ventilation, n (%)	7 (5.9)	2 (15.4)	5 (4.7)
High-flow nasal cannula oxygen therapy, n (%)	5 (4.2)	0 (0.0)	5 (4.7)
Vasopressor support, n (%)	41 (34.5)	6 (46.2)	35 (33.0)
Renal replacement therapy, n (%)	9 (7.6)	1 (7.7)	8 (7.5)
V-A ECMO, n (%)	0 (0.0)	0 (0.0)	0 (0.0)
V-V ECMO, n (%)	1 (0.8)	0 (0.0)	1 (0.9)
Acute respiratory distress syndrome, n (%)	6 (5.0)	1 (7.7)	5 (4.7)
Anticancer treatment during the ICU stay, n (%)	33 (27.7)	1 (7.7)	32 (30.2)
Life-sustaining treatment limitation in the ICU, n (%)	2 (1.7)	0 (0.0)	2 (1.9)
Total serum bilirubin at ICU discharge, micromol/L, median [IQR]	9.5 [6.0−20.8]	9.0 [6.3−12.8]	9.5 [6.0−20.8]
ECOG PS 3 or 4 after ICU discharge^a^, ^c^, n (%)	75 (65.2)	9 (75.0)	66 (64.1)
Detailed ECOG PS distribution			
0	1 (0.9)	0 (0.0)	1 (1.0)
1	39 (33.9)	3 (25.0)	36 (35.0)
2	39 (33.9)	2 (16.7)	37 (35.9)
3	25 (21.7)	3 (25.0)	22 (21.4)
4	11 (9.6)	4 (33.3)	7 (6.8)
ECOG PS change (before - after the ICU stay), median [IQR]	0 [0−1]	1 [0−2]	0 [0−1]

ECOG PS: Eastern Cooperative Oncology Group Performance Status; SAPS II: Simplified Acute Physiology Score version II; HM: hematological malignancy; ICU: intensive care unit; HSCT: hematopoietic stem-cell transplantation; V-A ECMO: veno-arterial extracorporeal membrane oxygenation; V-V ECMO: veno-venous extracorporeal membrane oxygenation.

a An ECOG PS of 3 or 4 defined poor performance status.

b Some patients had a history of more than one of the listed conditions.

c Available for 115 of the 119 patients.

Among the 13 patients who did not resume anticancer treatment, the reasons for not resuming treatment were persistent infection or excessive infectious risk (n = 4), disease progression leading to best supportive care (n = 3), treatment-related toxicity leading to ICU admission and subsequent decision not to reintroduce the implicated anticancer therapy (n = 2), poor general condition (n = 2), the patient's decision to forgo further anticancer treatment (n = 1), and cardiovascular comorbidity precluding further anticancer treatment (n = 1).

Of the 106 treated patients, 69 (65.1%) were given chemotherapy only and 21 (19.8%) combined chemotherapy and immunotherapy. The median time to treatment resumption was 18 days [interquartile range, 6.0–49.5]. The planned anticancer regimen was modified in 20/103 (19.4%) patients. These modifications consisted of drug changes in 11/20 patients, dosage reductions in 8 patients, and both in one patient. The reason or reasons for treatment modification were poor general health (n = 11), persistent infection (n = 6), comorbidities (n = 2), treatment-related toxicity (n = 2), and acute kidney injury (n = 1). A tumor response occurred in 102/106 (96.2%) patients including 81/106 (76.4%) with a complete response, 15/106 (14.2%) with a partial response, and 6/106 (5.7%) with stable disease.

By multivariable analysis (Table 3), cardiovascular disease was independently associated with a lower likelihood of anticancer treatment resumption (HR, 0.41; 95%CI, 0.20–0.84; *P* = 0.014), whereas diagnosis of the HM during the ICU stay was independently associated with a higher likelihood of treatment resumption (HR, 3.03; 95%CI, 1.53–6.01; *P* = 0.002).

**Table 3:** Multivariable analysis to identify factors independently associated with anticancer treatment resumption after discharge from the intensive care unit.

Variables	Multivariable analysis	
	HR (95%CI)	*P* value
Age at ICU admission	1.00 (0.98–1.01)	0.573
SAPS II	1.00 (0.98–1.01)	0.652
Infection	0.83 (0.46–1.51)	0.542
Cardiovascular disease	**0.41 (0.20–0.84)**	**0.014**
HM diagnosed in the ICU	**3.03 (1.53–6.01)**	**0.002**
Length of stay in the ICU	0.98 (0.95–1.01)	0.134
Vasopressor support	1.20 (0.77–1.86)	0.429
Anticancer treatment during the ICU stay	1.01 (0.54–1.89)	0.983
ECOG PS change (before - after the ICU stay)	0.82 (0.65–1.05)	0.112

HR: hazard ratio; 95%CI: 95% confidence interval; ICU: intensive care unit; SAPS II: Simplified Acute Physiology Score version II; ECOG PS: Eastern Cooperative Oncology Group Performance Status.

### Outcomes

**Fig. 2:**
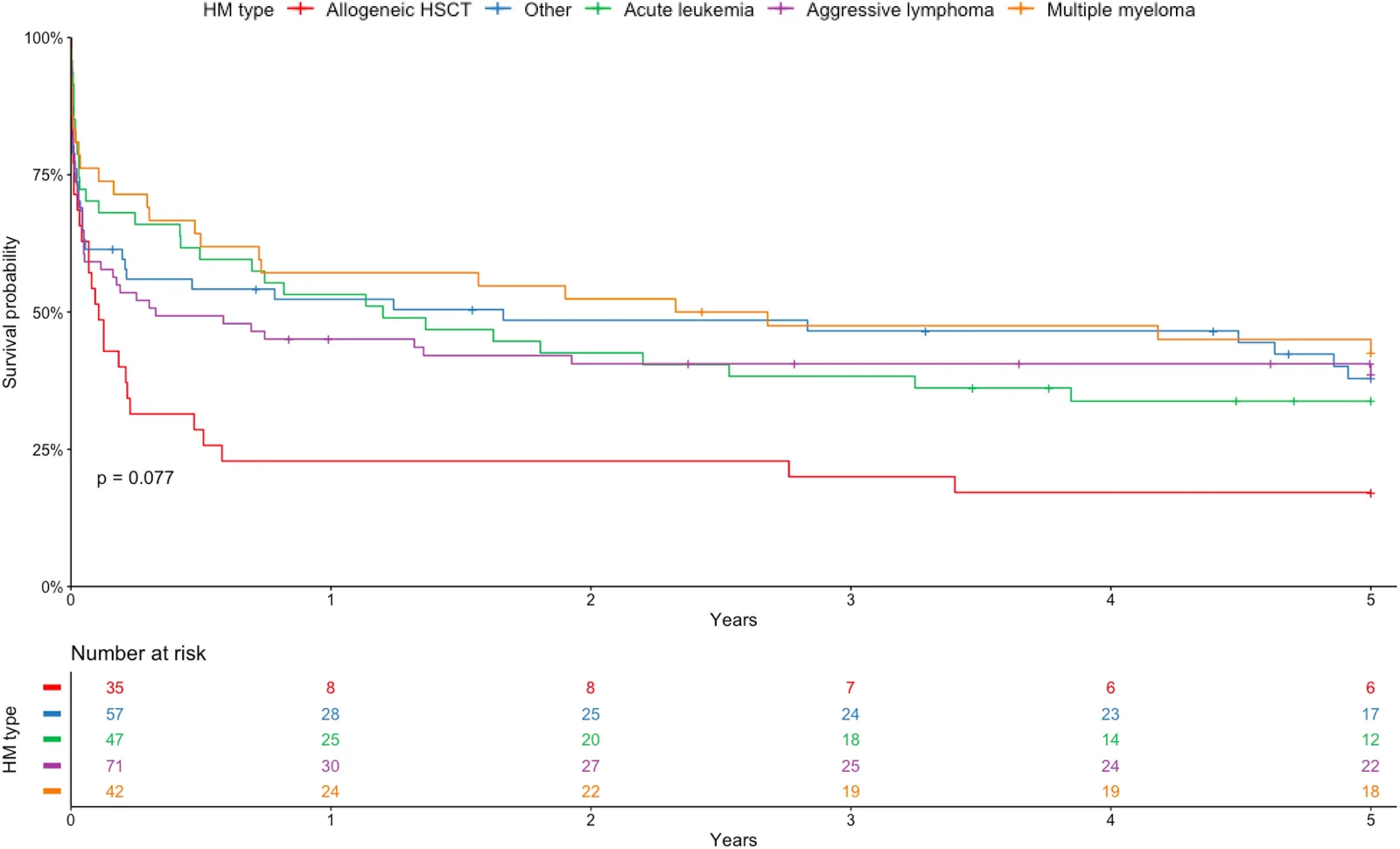
Kaplan–Meier curve of survival from intensive-care-unit admission (n = 252) to five-year follow-up. Survival beyond the acute phase of critical illness was substantial, with most deaths occurring during the ICU stay or within the first year after admission, and a marked attenuation of mortality thereafter. HM: hematological malignancy; HSCT: hematopoietic stem-cell transplantation.

The median follow-up was 8.7 years (interquartile range 6.8–10.2 years). In the overall cohort, survival rates 6 months and one, two, three, and five years after ICU admission were 50.0% (n = 126), 46.0% (n = 116), 41.3% (n = 104), 38.9% (n = 98), and 36.1% (n = 91), respectively (Fig. 1). Fig. 2 is the Kaplan–Meier curve for five-year survival in the overall cohort. Among the 177 patients who died during the entire follow-up period, the main causes of death were HM-related in 63 (35.6%) patients, infection in 51 (28.8%) patients, cardiac arrest in 11 (6.2%) patients, and specific anticancer treatment toxicity in 9 (5.1%) patients. At the time of death, 130 (73.4%) patients had active disease, whereas 87 patients (49.2%) were in remission and 34 patients (19.2%) were considered cured.

### Independent risk factors for mortality among ICU survivors

The multivariable analyses in the 171 ICU survivors used the allogeneic HSCT category as the reference (Table 4). The presence of one or more cardiovascular risk factors was independently associated with higher one-year mortality (HR, 1.78; 95%CI, 1.03–3.08; *P* = 0.038). The risk of death at 1 year was lower in patients with multiple myeloma (HR, 0.32; 95%CI, 0.13–0.77; *P* = 0.010) and in those classified as having other HMs (HR, 0.25; 95%CI, 0.10–0.65; *P* = 0.004).

**Table 4:** Multivariable analysis to identify factors associated with 1-year and 5-year mortality among ICU survivors.

Multivariable analysis of factors associated with one-year mortality		
Variables	HR (95%CI)	*P* value
SAPS II	1.01 (1.00–1.03)	0.158
Cardiovascular risk factors	**1.78 (1.03–3.08)**	**0.038**
HM types		
Allogeneic HSCT	REF	
Acute leukemia	0.41 (0.17–1.02)	0.054
Aggressive lymphoma	0.62 (0.29–1.32)	0.219
Multiple myeloma	**0.32 (0.13–0.77)**	**0.010**
Other HMs	**0.25 (0.10–0.65)**	**0.004**
HM diagnosed in the ICU	0.32 (0.07–1.35)	0.119
Highest number of organ failures	1.14 (0.90–1.44)	0.286
ECOG PS (before - after the ICU stay)	1.13 (0.87–1.46)	0.372

Multivariable analysis of factors associated with five-year mortality		
Variables	HR (95%CI)	*P* value
Infection	**0.54 (0.33–0.87)**	**0.011**
Cardiovascular disease	1.46 (0.86–2.49)	0.160
HM classification		
Allogeneic HSCT	REF	
Acute leukemia	0.68 (0.32–1.42)	0.306
Aggressive lymphoma	0.61 (0.31–1.22)	0.161
Multiple myeloma	0.49 (0.23–1.03)	0.061
Other HMs	**0.40 (0.19–0.86)**	**0.019**
HM diagnosed in the ICU	**0.31 (0.12–0.81)**	**0.018**
ECOG PS change (before - after the ICU stay)	1.20 (0.95–1.53)	0.124

HR: hazard ratio; 95%CI, 95% confidence interval; SAPS II: Simplified Acute Physiology Score version II; HM: hematological malignancy; HSCT: hematopoietic stem-cell transplantation; ECOG PS: Eastern Cooperative Oncology Group Performance Status.

Factors independently associated with a lower risk of five-year mortality were infection (HR, 0.54; 95%CI, 0.33–0.87; *P* = 0.011), HM diagnosed during the ICU stay (HR, 0.31; 95%CI, 0.12–0.81; *P* = 0.018), and classification as other HMs (HR, 0.40; 95%CI, 0.19–0.86; *P* = 0.019).

## Discussion

### Key findings

This retrospective observational study assessed anticancer treatment and five-year survival in patients with HMs who required ICU admission. Two-thirds of the patients were discharged alive from the ICU. Long-term survival was substantial, with more than a third of patients alive after five years. Nearly all ICU survivors with indications for further anticancer treatment were able to receive this treatment, although modifications of the initially planned regimen were required in about one-fifth of patients. Importantly, the factors independently associated with treatment resumption and long-term survival in ICU survivors were mainly related to the patients’ baseline characteristics and HM type rather than to the severity of the acute critical illness or intensity of organ support in the ICU.

### Comparison to previous studies

Overall, our population is similar to those in previous studies of critically ill patients with HMs. Demographic characteristics, HM subtypes, and the intensity of organ support during the ICU stay were comparable. In particular, the need for organ support was in line with previous reports, with 108 patients (43.0%) requiring mechanical ventilation, 133 (53.0%) vasopressor support, and 37 (14.7%) renal replacement therapy during the ICU stay. However, fewer patients had acute myeloid leukemia (19.0% vs. 25–35%) and more patients had an ECOG PS of 3–4 before ICU admission and after ICU discharge [7,16,18].

Among ICU survivors with indications for further anticancer treatment, nearly 90% were able to receive this treatment. This proportion was similar in two previous studies [7,18] and lower, at about 60%, in another [8]. These discrepancies may be related to differences in HM type and status among ICU survivors and in institutional organization and admission practices [19].

One-year mortality in our cohort was lower than in several previous studies [18,20,21]. The smaller proportion of patients with acute myeloid leukemia may have contributed to this difference, given the persistently high mortality in critically ill patients with acute leukemia [22,23]. A recent meta-analysis found a pooled ICU mortality rate of 52% in patients with acute leukemia [24]. However, the lower one-year mortality in our study is noteworthy since PS was markedly impaired both before and after the ICU stay, a characteristic well known to predict mortality in critically ill patients with malignancies [18,25]. The lower mortality may reflect improvements in the management of critically ill patients with HMs over time, as well as advances in anticancer therapies, which are increasingly effective and better tolerated, even in frail patients [6,26,27]. To our knowledge, few studies have evaluated survival beyond the first year after ICU admission. Importantly, most deaths occurred within this first year. Subsequently, mortality was substantially lower, possibly reflecting the effectiveness of post-ICU oncological management. Thus, among the patients given post-ICU anticancer treatment, 96.2% achieved a tumor response and 76.4% a complete tumor response.

Interestingly, our findings are consistent with previous reports showing that treatment resumption and long-term mortality in ICU survivors are chiefly associated with patient-related factors, including comorbidities and HM characteristics, rather than with the intensity of organ support [7,8,28]. Cardiovascular comorbidities were independently associated with both the likelihood of treatment resumption and long-term survival. A likely explanation is that cardiovascular disease limits anticancer treatment options. Among treatments that have radically improved the prognosis of HMs, several carry substantial cardiovascular toxicities and may not be tolerated by patients with cardiovascular disease [[29], [30], [31], [32]]. Furthermore, cardiovascular comorbidities themselves may be an independent determinant of mortality. Whether other organ dysfunctions may similarly influence post-ICU oncological management remains uncertain. Acute kidney injury occurring during the ICU stay was not associated with subsequent anticancer treatment resumption in our cohort. Although this finding may partly reflect limited statistical power, similar results have been reported previously [7]. However, caution is warranted when interpreting this apparent lack of association, as detailed data regarding renal recovery and persistent kidney dysfunction after ICU discharge were unavailable both in our study and in previous reports addressing treatment resumption. Acute kidney injury has been associated with impaired treatment response and increased mortality in patients with hematological malignancies and may restrict the delivery of optimal anticancer therapy [33]. The cause of critical illness may also contribute to long-term prognosis. Infection as the reason for ICU admission was independently associated with lower five-year mortality. Infection is usually reversible and may not affect HM control, whereas HM-related events may predict poorer oncological outcomes.

### Study implications

This study adds to the extensive data on the short-term post-ICU prognosis in patients with HMs [18,[34], [35], [36]] by focusing on anticancer treatment resumption and five-year survival. Our finding that HM characteristics and cardiovascular status were the main factors associated with long-term oncological outcomes in survivors should be considered when devising ICU-admission policies. Furthermore, close collaboration between intensivists and hematologists is clearly crucial when making ICU admission decisions and planning the post-ICU management.

Concern has been raised that greater care intensity may be associated with worse post-intensive care syndrome and, therefore, with worse oncological outcomes. In our study, however, the intensity of ICU care was not associated with treatment resumption or long-term mortality in survivors. PS was significantly worse after than before the ICU stay but, in the adjusted analysis, the change was not independently associated with treatment resumption or mortality. Most of the ICU survivors in our study were able to resume anticancer treatment and to achieve meaningful tumor responses. Thus, concern that high-intensity care might jeopardize post-ICU anticancer treatment may be unwarranted. This point deserves careful attention when considering limitations to life-sustaining treatments.

### Strengths and limitations

This study has several strengths. The general characteristics of our cohort were consistent with previous reports and the patients had substantial critical illness severity that often required organ support, supporting the external validity of our findings. Anticancer treatment resumption is a pragmatic and clinically meaningful endpoint. Finally, the five-year follow-up adds substantially to the previously available outcome data for critically ill patients with HMs.

One limitation of our study is the single-center recruitment, which may limit the generalizability of our findings. Second, the retrospective study design is associated with inherent biases, including potential residual confounding. Third, the absence of an association between markers of ICU care intensity and long-term outcomes should be interpreted with caution. The limited number of patients who did not resume anticancer treatment may have limited the statistical power to identify associations between ICU-related variables and long-term outcomes. In addition, although ECOG PS was available before and after the ICU stay, more detailed assessments of post-intensive care syndrome—including physical, cognitive, and psychological impairments—as well as persistent organ dysfunction were not available. Consequently, the influence of post-ICU sequelae on subsequent oncological management may not have been fully captured. Furthermore, only a history of cardiovascular risk factors and cardiovascular disease at ICU admission was collected. Detailed information regarding cardiovascular events occurring during or after the ICU stay and persistent cardiovascular dysfunction was not collected. Therefore, the mechanisms explaining the association between cardiovascular disease and subsequent oncological management could not be investigated. Finally, the patients were included between 2014 and 2019, a period during which treatments for HMs evolved substantially. As the fast pace of treatment advances continues, outcomes may change over time, potentially limiting the applicability of our results to current practice.

## Conclusion

In our cohort of critically ill patients with HMs, ICU survival was substantial, and nearly all ICU survivors were able to resume anticancer treatment if indicated. The intensity of organ support during the ICU stay was not independently associated with long-term oncological treatment or survival in ICU survivors. Instead, baseline cardiovascular disease and HM type were the principal determinants of both treatment resumption and long-term survival. Thus, when making decisions for critically ill patients with HMs based on anticipated long-term outcomes, critical illness severity and care intensity should be considered alongside comorbidities and disease-specific outcomes rather than in isolation.

## Authors’ contributions

OM, JMT and SB designed the study and wrote the manuscript. OM, MG, BP, and SB collected the data. SB and AM performed the statistical analysis. AM, JBM, JR, and EC revised the manuscript.

## Consent for publication

Not applicable

## Ethics approval and consent to participate

This work was approved by our institutional ethics committee (#20.02). All ICU survivors were informed in writing about the study and were given the option to decline participation.

## Funding

None.

## Availability of data and material

The datasets used and/or analyzed during the current study are available from the corresponding author on reasonable request.

## Declaration of competing interest

None of the authors has any conflicts of interest in relation to this work.
